# The First Report of Mycoplasmas in Antarctic Pinnipeds: The Results of a Survey

**DOI:** 10.3390/ani15070937

**Published:** 2025-03-25

**Authors:** Orestes M. Vega-Orellana, Rubén S. Rosales, José B. Poveda, Francisco J. García-Peña, Daniel García-Párraga, Susana Pedraza-Díaz, Luis M. Ortega-Mora, Joachim Spergser, Ana S. Ramírez

**Affiliations:** 1 Unidad de Epidemiología y Medicina Preventiva, Instituto Universitario de Sanidad Animal y Seguridad Alimentaria—IUSA, Universidad de Las Palmas de Gran Canaria, Campus Montaña Cardones, 35416 Arucas, Spain; orestesvega@gmail.com (O.M.V.-O.); jose.poveda@ulpgc.es (J.B.P.); anasofia.ramirez@ulpgc.es (A.S.R.); 2 Laboratorio Central de Veterinaria de Algete, 28110 Madrid, Spain; fgarciap1960@gmail.com; 3 Veterinary Services, Oceanografic, Ciudad de las Artes y las Ciencias, 46013 Valencia, Spain; dgarcia@oceanografic.org; 4 National Center for Environmental Health (CNSA), Instituto de Salud Carlos III, 28220 Majadahonda, Spain; spedraza@isciii.es; 5 SALUVET, Animal Health Department, Faculty of Veterinary Sciences, Universidad Complutense de Madrid, 28040 Madrid, Spain; luis.ortega@vet.ucm.es; 6 Department of Pathobiology, Institute of Microbiology, University of Veterinary Medicine, A-1210 Vienna, Austria; joachim.spergser@vetmeduni.ac.at

**Keywords:** mycoplasma, marine mammal, seal, Antarctic fur seal, Weddell seal, Southern elephant seal, phocid, otariid, pinniped, Antarctica

## Abstract

The aim of this study was to investigate the presence of *Mycoplasma* spp. and their identification in seals from Antarctica. During January and February 2010, 59 Antarctic fur seals (*Arctocephalus gazella*), 17 Weddell seals (*Leptonychotes weddellii*), and 5 Southern elephant seals (*Mirounga leonina*) were captured in three Antarctic islands. Oral and genital samples were collected, cultured, and cloned. The Intergenic Spacer Region 16S-23S rDNA (ISR) PCR products were sent for sequencing. Seventy-four (91.4%) out of the eighty-one seals sampled were PCR positive. From those, 57 isolates were cultured. Mycoplasmas were more prevalent in the mouth in comparison to the reproductive tract. The percentage of isolates were 76.3%, 58.8%, and 40.0% for Antarctic fur seals, Weddell seals, and Southern elephant seals, respectively. The ISR sequences divided the isolates into six clusters. Four clusters presented a very high similarity percentage with mycoplasma sequences obtained from seals. However, none of these mycoplasmas have been described to date. Cluster 1 is also close to *M. miroungigenitalium*. This study represents the first report of *Mycoplasma* species adapted to Antarctic pinnipeds. The findings contribute to the understanding of the ecology of mycoplasmas in Antarctic pinnipeds.

## Simple Summary

This study investigates the presence of a type of bacteria called mycoplasmas in seals living in Antarctica. In January and February 2010, researchers collected samples from 81 seals on three Antarctic islands. These samples were taken from the animals’ mouths and reproductive organs and were analyzed by sequencing a region of the genome. The results showed that mycoplasmas are very prevalent in Antarctic seals and six different *Mycoplasma* species were isolated. Two of them may represent new species of this type of microorganism. The other four are very closely related to other uncharacterized mycoplasma isolates from other types of seal. The findings provide new insights into the microbial communities of Antarctic seals and suggest that mycoplasmas are part of their microbiota.

## 1. Introduction

Antarctica is one of the most unspoiled regions on Earth, with a biodiversity that is much more diverse, ecologically complex, and geographically structured than previously thought. The wildlife of Antarctica is truly unique, comprising a remarkable collection of endemic and resident species, along with seasonal migrants [1]. The Antarctic region is zoologically and geographically isolated, with Antarctic pinnipeds being one of the most representative groups in the Antarctic ecosystem. Six pinniped species (seals, fur seals, and sea lions) commonly inhabit the Antarctic Peninsula region: the phocids Weddell seal (*Leptonychotes weddellii*), Southern elephant seal (*Mirounga leonina*), crabeater seal (*Lobodon carcinophaga*), leopard seal (*Hydrurga leptonyx*), and Ross seal (*Ommatophoca rossii*), and the only species of otariid in the Southern Ocean, the Antarctic fur seal (*Arctocephalus gazella*) [2,3].

*Mycoplasma* is a genus of class *Mollicutes*, which comprises a group of wall-less prokaryotes and are among the smallest self-replicating organisms. All mollicutes are commensals or parasites of animal, insect, and plant hosts [4]. The presence and effects of these microorganisms in terrestrial animals, plants, and humans have been extensively studied; however, studies in pinnipeds are limited. Several mycoplasmas have been isolated from diseased Harbor Seals (*Phoca vitulina*). *Mycoplasma* (*M.*) *phocae* were isolated from animals that died of epizootic pneumonia that was attributed to an influenza virus in 1979–1980 along the east coast of the United States, while *M. phocicerebrale* and *M. phocirhinis* were detected during the seal epidemic in the North Sea and in the Baltic Sea in 1988 [5,6,7]. Volokhov et al. (2022) isolated *M. miroungirhinis* and *M. miroungigenitalium* from Northern elephant seals (*Mirounga angustirostris*), *M. phocoenae* from Harbour porpoise (*Phocoena phocoena*), and *M. phocoeninasale* from Harbour porpoise and California sea lions (*Zalophus californianus*) from the west coast of the United State [8].

Studies of the bacterial populations present in pinnipeds can be useful to know more about the biology and ecology of these organisms. *M. phocicerebrale* was demonstrated to be zoonotic when it was associated with the seal-finger condition after human interaction with seals [9]. Recently, another mycoplasma has been related to the same condition, *M. phocimorsus* [10]. However, most reports are from the northern hemisphere, although some clinical presentations resembling seal finger have been reported in the southern hemisphere without discovering the pathogen involved [11]. To our knowledge, there are no reports on the isolation of mycoplasmas from pinnipeds in the Antarctic region.

Because of the increasing number of anthropogenic activities in Antarctica related to tourists and research scientists [11] and the lack of knowledge about the presence of mycoplasmas in pinnipeds, the aim of the present study was to investigate the presence of mycoplasmas in three pinniped species from Antarctica, and to identify mycoplasma species with the potential to cause disease in humans.

## 2. Materials and Methods

### 2.1. Sample Collection

In January and February 2010, a total of 81 seals were captured at Deception Island (63°00′ S 60°40′ W), Rongé Island (64°43′ S 62°41′ W), and Avian Island (67°46′ S 68°43′ W), Antarctica. For the collection of samples, animals were randomly selected, captured, and physically restrained according to standard procedures, with all seal handling conducted under scientific authorizations and permission granted by the Spanish Polar Committee (CMT2008-00570), in compliance with the Antarctic Treaty System. Each animal was tagged to prevent duplicate sampling. The tagging of animals was performed using plastic tags of different colors according to the campaign year, featuring an identification number and a contact email address. The placement of the tag varied depending on the animal species (right pectoral flipper or caudal fin). The study included 59 Antarctic fur seals (*A. gazella*) from the Otariidae family, 17 Weddell seals (*L. weddellii*), and 5 Southern elephant seal (*M. leonina*), both from the Phocidae family (Table 1). All animals, including adults and subadults, exhibited good physical condition and showed no clinical symptoms at the time of sampling. For the investigation of *Mycoplasma* presence in the oral and genital tracts, samples were collected by directly inserting sterile cotton swabs, which were then placed in FBP medium [12] containing 0.5% activated charcoal (Sigma Ltd., Madrid, Spain) and stored at −20 °C until they were cultured in the laboratory.

### 2.2. Mycoplasma Isolation and Identification

#### 2.2.1. Mycoplasma Isolation

Samples were thawed at room temperature, vortexed and inoculated into standard mollicutes broth and agar media [13], and incubated at 37 °C up to two weeks. After that time and after DNA extraction from broth, a mycoplasma-specific PCR [14] was used to screen all the cultures. Positive cultures were kept at −80 °C until mycoplasma identification.

Positive samples were subcultured into liquid SP4-II medium [15] at 37 °C for one day. After the incubation, the cultures were filtered through 0.45 µm pore-size sterile membranes (Acrodisc^®^ Syrynge Filters, Pall Corporation, Madrid, Spain) into the same medium. When a color change or turbidity of the medium was observed, cultures were plated onto SP4-II medium agar [15] and incubated at 37 °C under aerobic conditions. Plates were observed daily with a microscope for colonies growth. Samples that did not show growth after two weeks were considered negative. Pure cultures were obtained using triple filter cloning [16].

#### 2.2.2. DNA Extraction and PCR

DNA was extracted from cloned cultures using REALPURE Spin Kit (Real, Durviz S.L, Valencia, Spain). Generic Mycoplasma primers for conventional PCR described by Botes et al. (2005) [17] and applied to real-time PCR [18] were used for mycoplasma confirmation. Additionally, the intergenic space region (ISR) between 16S and 23S rRNA genes was amplified by conventional PCR, as published previously [19]. Furthermore, 16S rRNA gene electrophoretic patterns were studied to compare the isolates using 16S-rDNA-PCR-DGGE (Denaturing Gradient Gel Electrophoresis) [20].

#### 2.2.3. Sequencing and Sequence Analysis

All ISR PCR products were purified with Cycle Pure Kit (Omega Bio-Tek, Inc., Norcross, GA, USA) before sending it for sequencing elsewhere. Sequencing was conducted by Macrogen Europe (Amsterdam, The Netherlands) using an Applied Biosystems 3730xl DNA Analyzer. Both strands of ISR DNA were sequenced at least twice. The resulting chromatograms were examined, and a consensus sequence were produced from analysis of the forward- and reverse-complemented sequences by the software Finch Version 1.4.0 (Geospiza Inc., Seattle, WA, USA). BioEdit 7.2.5 [21] was used for pairwise aligning the sequences and for calculating sequence similarities. All obtained ISR sequences were analyzed using the BLAST (https://blast.ncbi.nlm.nih.gov/Blast.cgi, accessed on 17 March 2025) algorithm [22] at the National Center for Biotechnology Information (NCBI). For molecular analyses and the creation of dendrograms, MEGA version 11 [23] was used. The dendrograms were compared with phylogenetic groups and clusters based on the 16S rRNA molecule [24,25,26].

## 3. Results

### 3.1. Mycoplasma Detection and Isolation

Mycoplasmas were detected and isolated from all three seal species sampled in this study. The detection of mycoplasmas was high before cloning. More detailed results of the study are shown in Table 2 and Supplementary Table S1. Seventy-four out of eighty-one seals (91.4%) tested positive using generic mycoplasma PCR [14]. Of these, 62 oral samples (76.5%) and 21 genital samples (25.9%) were positive. Nine (11.1%) animals gave a positive result in both types of samples. Primary cultures were achieved in 69 out of the 74 (93.2%) PCR positive seals or 85.2% (69/81) from the sampled ones. The percentages from the type of sample (oral or genital) primary isolation and positive by mycoplasma PCR were 100% (62/62) from oral samples and 57.1% (12/21) from genital samples. Primary mycoplasma cultures from both sites were seen in five seals.

Normally, one type of mycoplasma was isolated in pure culture per animal, either from an oral or genital sample, with the exception of two animals. In both cases, the mycoplasma isolated from the oral sample was different to the one isolated from the genital tract, as can be seen in Table 2 (seals AV-11 and DE-14). Pure mycoplasma isolates were obtained from 55 seals, 67.9% (55/81) from the 81 seals, 74.3% (55/74) from mycoplasma positive PCR seals, or 79.7% (55/69) from primo-culture-positive seals. All the isolates produced the typical fried-egg colony morphology. Information about the host species and location can be seen in Table 1. The only animal sampled in Rongé Island was a Weddell seal from which one isolate was obtained. Of the 20 phocids sampled in Avian Island, 10 isolates from nine seals (45%) were obtained (Table 1). At Deception Island, 45 mycoplasmas isolates from 44 seals out of 59 (74.6%) were achieved. Related to the host species, the percentage of mycoplasma isolation was 76.3%, 58.8%, and 40.0% for Antarctic fur seals, Weddell seals, and Southern elephant seals, respectively.

### 3.2. Mycoplasmas Identification and Sequencing

All isolates were confirmed as mollicutes by real-time PCR [18]. ISR-amplified fragments yielded a single PCR product. Based on the length of the PCR products, at least three groups were detected (A, B and C) (Table 3). When the DGGE was performed with the same samples, four profiles (A’, B’, C’ and D’) were observed, but with the combination of ISR groups and DGGE profiles, five groups were discovered (AA’, AC’, AD’, BB’ and CB’). However, by sequencing the ISR, strains were divided into six clusters. The length of the ISRs varied from 246 to 326 bp (Table 3). Strains within five clusters showed identical ISR sequences. In Cluster 3, 50% of the strains presented an extra Adenine (A) in a poly-A region at position 201, presenting similarities between 99.6 and 100%. Both strains of Cluster 4 present an extra A in just one of the operons in a poly-A region at position 72. Table 3 shows the six mycoplasma clusters found in pinnipeds captured in three islands from Antarctica. Similarity values among their ISR ranged from 39.5% between Clusters 1 and 2 to 83.4% between Cluster 2 and 4. ISR sequences were sent to GenBank and the given accession numbers are shown in Table 2.

The mycoplasma strains with the highest ISR similarities are shown in Table 3. All the closest related mycoplasmas have not been validly described.

Table 4 displays the cluster distribution of mycoplasmas found in pinnipeds divided by sample site (oral versus reproductive tract). There are more *Mycoplasma* isolates from oral samples than from the genital ones. *Mycoplasma* strains from Clusters 1 and 3 were detected exclusively in Antarctic fur seals (*A. gazella*), the first one just in genital samples and the latter in both sites, oral and genital (Table 4). Mycoplasmas from Cluster 4 were isolated from oral samples from Weddell seals (*L. weddellii*). Members of Clusters 5 and 6 were recovered from two of the three pinnipeds, while mycoplasmas of Cluster 2 were found in all three pinnipeds and from the mouth and genitals.

### 3.3. Dendrograms

**Figure 1:**
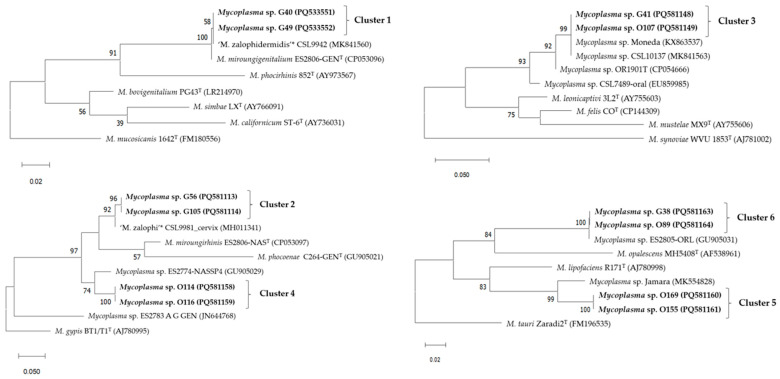
Dendrograms showing the mycoplasmas with highest similarities based on the 16S to 23S rDNA ISR sequences within Clusters 1 to 6 based on the data obtained in the present study. The Maximum Likelihood method and Tamura–Nei model with 1000 bootstrap replicates was used in MEGA 11.0 [23]. Bootstrap percentage values are located at nodes of the tree. GenBank accession numbers are indicated for each strain in parentheses. Names in bold letters represent a selection of relevant mycoplasma isolates from this study. The letter ‘^T^’ at the end of strain names indicates strain types. The bars below each tree indicate substitutions per site.* Undescribed *Mycoplasma* species.

The resulting dendrograms from the ISR sequences are displayed in Figure 1. All the isolates obtained in this study were located within the *Hominis* group of genus *Mycoplasma*. Clusters 1, 5, and 6 are included in the *M. bovis* cluster, Clusters 2 and 4 belong to the *M. hominis* cluster, and Cluster 3 is positioned within the *M. synoviae* cluster.

## 4. Discussion

This is the first report of mycoplasmas in pinnipeds from Antarctica. In this study, we described the detection and isolation of mycoplasmas from three apparently healthy Antarctic pinniped species sampled in three islands along the west coast of the Antarctic Peninsula. Seals sampled were Antarctic fur seals (59), Weddell seals (17), and Southern elephant seals (5). Other Antarctic mammals of interest include the crabeater seal and the leopard seal; however, their non-gregarious behavior and preference for remaining on ice floes hindered their capture and sampling [27]. Mycoplasmas were detected in 91.4% of the seals, with a prevalence three times higher (76.5 vs. 25.9%) in the oral cavity than in the genital tract. Nonetheless, the number of cloned mycoplasmas isolated decreased to 67.9%. The percentages of mycoplasma isolates were 76.3% for Antarctic fur seals, 58.8% for Weddell seals, and 40.0% for Southern elephant seals. Mycoplasma strains were recovered from 48 oral and 9 genital samples. These results are in accordance with the data obtained from other seal species, where mycoplasmas could be isolated from nasal and genital samples [8] and from 72% or 37.9% of nasal cavities [28,29], indicating that mycoplasmas could be considered as common microorganisms of the microbiome of seals. However, further studies, such as metagenomics analysis, should be performed to confirm this finding.

After analyzing the ISR, six clusters of mycoplasmas were identified. The closest relatives after BLAST search were other seal mycoplasmas in five of the six clusters. In contrast, members of Cluster 5 are related to a mycoplasma strain (Jamara, MK554828) derived from a dromedary, although the similarity percentage is below 93%. Four of the clusters presented a very high similarity percentage with mycoplasma sequences obtained from seals. Cluster 1 members present 99.69% similarity with ‘M. zalophidermidis’ (DQ840512) and Cluster 2, 98.80% similarity to ‘M. zalophi’ (MH011341), both isolated from California sea lions. However, none of these mycoplasmas have been validly described up to date, although an attempt was published for ‘M. zalophi’ [29]. Cluster 3 ISR sequences were shown to be identical to the ISR of *Mycoplasma* strain CSL10137 (MK841563) isolated also from California sea lion and to the ISR of *Mycoplasma* strain Moneda recovered from a zoo-kept South American sea lion. Cluster 6 has a 99.39% similarity with *Mycoplasma* sp. ES2805-ORL (GU905031) from a Northern elephant seal. Cluster 4 presents the lowest similarity value (90.80%) to a seal mycoplasma, namely the strain ES2774-NASSP4 (GU905029) isolated from a Northern elephant seal. The *M. miroungigenitalium* ES2806-GEN^T^ (CP053096) ISR is also very similar to Cluster 1 ISRs (99.08%) and to the ISR of ‘M. zalophidermidis’ (98.77%); however, based on complete genome analysis, the latter strain appears to be a distinct species from *M. miroungigenitalium* [8]. Although we cannot definitively identify the isolates using ISR analysis, we can conclude that the isolates in clusters 4 and 5 are unique, with this being the first time they have been isolated. Nevertheless, further studies should be performed in order to fully characterize these isolates. The other four clusters are very closely related to other seal mycoplasmas.

Interestingly, mollicutes commonly found in non-Antarctic pinnipeds, like *M. phocae*, *M. phocirhinis*, and *M. phocicerebrale* [5,7,28,30], were not detected in our study, possibly indicating that these species do not occur in Antarctic pinnipeds. Cluster 1 isolates and ‘M. zalophidermidis’ have both been found in otariids and just from genital samples. Previous studies have detected ‘M. zalophi’ and *Mycoplasma* sp. closely related to Cluster 3 in the respiratory tract of otariids, such as the Australian fur seal (*Arctocephalus pusillus doriferus*) [28], and the California sea lion [29], but not in any species of family Phocidae. Notably, isolates from Cluster 3 were only found in otariids. On the contrary, isolates from Cluster 2, with a very high similarity to ‘M. zalophi’, have been isolated not only from otariids but also from phocids, such as Weddell seals and Southern elephant seals. Although most of the mycoplasmas were isolated from oral samples in both clusters, there are also few isolates from the genitals. Conversely, the *Mycoplasma* strain Mirounga ES2805-ORL (GU905031) (close to Cluster 6) was detected exclusively in the Northern elephant seal (Phocidae), and not in any otariids [31]. However, there are only two isolates from Cluster 6, one from the oral cavity of an otariid and the other from the genitals of a phocid.

Additionally, mycoplasmas have also been detected by metagenomic analysis in fecal samples from two Australian otariids, the Australian sea lion (*Neophoca cinerea*) [32], the Australian fur seal [33], as well as in Mexican phocids, Northern elephant seals, and Pacific harbor seals (*Phoca vitulina richardii*) [34]. In contrast, a study on the colonic microbiota of Arctic and Sub-Arctic seals failed to detect *Mollicutes* species [35]. In addition, *Mycoplasma* spp. were detected by PCR in nasal discharge samples from the following otariids: South American fur seal (*Arctocephalus australis*) [36] and Galapagos sea lions (*Zalophus wollebaeki*) [37].

The occurrence of these mollicutes may be influenced by differences in pinniped species or even taxonomic families (Otariidae and Phocidae), affecting their distribution. In our study, *Mycoplasma* spp. have been isolated from otariids more often than from phocids. This is in accordance with seal mycoplasmas isolated or detected in the southern hemisphere [28,32,33,36,37], contrary to the findings described for the northern hemisphere, where the detection and/or isolation of mycoplasmas is more prevalent in phocids [5,6,8,30,34].

The role of mollicutes has been extensively described in terrestrial vertebrates. They can be commensal species that do not harm the host or pathogenic species that cause diseases or contribute to their development [38]. However, the role of mycoplasmas in pinnipeds remains unclear. Although *Mycoplasma phocae*, *M. phocirhinis*, and *M. phocicerebrale* have been associated with mass-mortality events in Harbour seals, various authors suggest that these mycoplasmas are not primary pathogens but may act as opportunistic organisms, contributing to the development of disease [5,6,39]. Similarly, ‘M. zalophi’ has occasionally been associated with subdermal and muscle injuries, as well as arthritis and lymphadenopathy in California sea lions [29]. Also, *Mycoplasma* spp. were recovered from Galapagos sea lions showing respiratory disease, lethargy, and poor body conditions [37] as well as from the lungs of aborted Australian fur seal foetuses [28]. Conversely, the seals in our study were apparently healthy, indicating that these mycoplasmas are part of the normal bacterial microbiota of these species, as has been suggested for mycoplasmas detected in healthy South American fur seals [36] and Australian sea lions [28]. This article is a revised and expanded version of a paper entitled Mollicutes Found in Pinnipeds from Deception Island (Antarctica), which was presented at the 20th Congress of the International Organization for Mycoplasmology, Blumenau, Brazil, 1-6 June 2014 and included an analysis of a smaller sample size [40].

## 5. Conclusions

This study provides the first report of mycoplasmas in Antarctic pinnipeds, identifying several clusters and suggesting the presence of previously unidentified species. The information gathered contributes to the understanding of the ecology of mycoplasmas in Antarctic pinnipeds and will be valuable for future studies in this remote region. However, the role of these bacteria in pinnipeds’ health remains unclear, warranting further research.

## Figures and Tables

**Table 1:** The distribution of the samples vs. mycoplasma isolates by host species and location.

	Antarctic Fur Seals (*A. gazella*)	Weddell Seals (*L. weddellii*)	Southern Elephant Seal (*M. leonina*)	Total
Rongé Island	-	1/1 *	-	1/1
Avian Island	14/9	2/0	4/1	20/10
Deception Island	45/36	14/9	1/1	60/45
Total	59/45	17/10	5/2	81/57

* Number of animals sampled/number of mycoplasma isolates.

**Table 2:** Seal sampling and *Mycoplasma* isolates.

Sample ID	Island *	Host **	Mycoplasma Strain ***	Mycoplasma Cluster	ISR Accession Number	Sample ID	Island *	Host **	Mycoplasma Strain ***	Mycoplasma Cluster	ISR Accession Number
RG-1	R	*Lw*	G38	6	PQ581163	DE-24	D	*Ag*	O139	2	PQ581129
AV-2	A	*Ag*	G40	1	PQ533551	DE-25	D	*Ag*	O140	2	PQ581130
AV-3	A	*Ag*	G41	3	PQ581148	DE-26	D	*Ag*	O141	3	PQ581154
AV-6	A	*Ag*	O44	2	PQ581115	DE-29	D	*Lw*	O155	5	PQ581161
AV-10	A	*Ml*	O48	2	PQ581116	DE-31	D	*Lw*	O157	2	PQ581131
AV-11	A	*Ag*	G49 O49	1 2	PQ533552 PQ581117	DE-33	D	*Lw*	O159	2	PQ581132
AV-12	A	*Ag*	G50	1	PQ533553	DE-34	D	*Ag*	O160	2	PQ581133
AV-17	A	*Ag*	G55	1	PQ533554	DE-35	D	*Ag*	O161	2	PQ581134
AV-18	A	*Ag*	G56	2	PQ581113	DE-36	D	*Ag*	O162	2	PQ581135
AV-20	A	*Ag*	O58	2	PQ581118	DE-37	D	*Ag*	O163	2	PQ581136
DE-2	D	*Ag*	G105	2	PQ581114	DE-38	D	*Ag*	O164	2	PQ581137
DE-3	D	*Ag*	O106	2	PQ581119	DE-40	D	*Ag*	O166	2	PQ581138
DE-4	D	*Ag*	O107	3	PQ581149	DE-41	D	*Ag*	O167	3	PQ581155
DE-6	D	*Ag*	O109	2	PQ581120	DE-42	D	*Ag*	O168	2	PQ581139
DE-8	D	*Ag*	O111	2	PQ581121	DE-43	D	*Lw*	O169	5	PQ581160
DE-10	D	*Lw*	O113	2	PQ581122	DE-44	D	*Lw*	O170	2	PQ581140
DE-11	D	*Lw*	O114	4	PQ581158	DE-46	D	*Ml*	O172	5	PQ581162
DE-12	D	*Ag*	O115	3	PQ581150	DE-47	D	*Ag*	O173	2	PQ581141
DE-13	D	*Lw*	O116	4	PQ581159	DE-50	D	*Ag*	O176	2	PQ581142
DE-14	D	*Ag*	G129 O129	1 3	PQ533555 PQ581151	DE-51	D	*Ag*	O177	2	PQ581143
DE-15	D	*Ag*	O130	2	PQ581123	DE-52	D	*Ag*	O178	2	PQ581144
DE-16	D	*Ag*	O131	3	PQ581152	DE-53	D	*Ag*	O179	2	PQ581145
DE-17	D	*Ag*	O132	2	PQ581124	DE-54	D	*Ag*	O180	2	PQ581146
DE-18	D	*Ag*	O133	2	PQ581125	DE-55	D	*Ag*	O181	3	PQ581156
DE-19	D	*Ag*	O134	2	PQ581126	DE-56	D	*Ag*	O182	3	PQ581157
DE-20	D	*Ag*	O135	2	PQ581127	DE-58	D	*Ag*	O184	2	PQ581147
DE-21	D	*Ag*	O136	3	PQ581153	DE-89	D	*Ag*	O89	6	PQ581164
DE-22	D	*Lw*	O137	2	PQ581128						

* R: Rongué Island; A: Avian Island; D: Deception Island. ** *Lw*: *Leptonychotes weddellii*; *Ag*: *Arctocephalus gazella*; *Ml*: *Mirounga leonina*. *** G: genital sample; O: oral sample.

**Table 3:** Mycoplasma clusters, ISR length, and % similarity.

Cluster * n°	Group ** ISR	Group ** DGGE	ISR bp	(% Similarity) Mycoplasma
1	A	A’	324	(99.69%) ‘M. zalophidermidis’ *** (DQ840512) (99.08%) *M. mirungigenitalium* (CP053096)
2	B	B’	250	(98.80%) ‘M. zalophi’ *** (MH011341)
3	C	B’	296–297	(100%) *M.* sp. strain CSL10137 (MK841563)
4	B	B’	246	(90.80%) *M.* sp. Mirounga ES2774-NASSP4 (GU905029)
5	A	C’	318	(92.24%) *M*. sp. strain Jamara (MK554828)
6	A	D’	326	(99.39%) *M.* sp. Mirounga ES2805-ORL (GU905031)

* Based on the ISR sequences. ** Grouping based on the length and electrophoretic profile of PCR products. *** Undescribed *Mycoplasma* species.

**Table 4:** Cluster distribution of mycoplasmas found in pinnipeds and divided by the oral or genital sample.

Pinnipedia		N° Specimens			Mycoplasma Clusters ^4^						
Family	Species	Total	Positives ^1^	Cloned Cultures ^2^	1	2	3	4	5	6	Total
Otariidae	*A. gazella*	59	55	45	0/5 ^3^	27/2	9/1			1/0	37/8
Phocidae	*L. weddellii*	17	17	10		5/0		2/0	2/0	0/1	9/1
	*M. leonina*	5	3	2		1/0			1/0		2/0

^1^ Positive PCR using the generic mycoplasma primers [14]. ^2^ Positive in real-time PCR [17,18]. **^3^** Number of isolates from oral/genital origin. ^4^ Mycoplasma identification was based in the similarity of an ISR sequence with a known species using the BLASTn program.

## Data Availability

The original contributions presented in this study are included in the article/Supplementary Material. Further inquiries can be directed to the corresponding author.

## Supplementary Materials

The following supporting information can be downloaded at: https://www.mdpi.com/article/10.3390/ani15070937/s1, Table S1. Seal sampling and *Mycoplasma* isolates.
