# Supplementary material for: The First Report of Mycoplasmas in Antarctic Pinnipeds: The Results of a Survey
Source: Animals (Basel). 2025 Mar 25;15(7):937. doi: 10.3390/ani15070937 (PMC11987810; doi:10.3390/ani15070937)
Supplement: Supplementary file 1 [file animals-15-00937-s001.zip › animals-3469320-supplementary.pdf]

Table S1. Seal sampling and *Mycoplasma* isolates.

Sample ID	Island	Host	PCR (oral/genital)	Primary culture (oral/genital)	Mycoplasma strain*	Mycoplasma cluster	ISR Accession number
RG-1	Rongé	*L. weddellii*	+/+	+/+	G38	6	PQ581163
AV-1	Avian	*A. gazella*	-/-	-/-	-	-	-
AV-2	Avian	*A. gazella*	-/+	-/+	G40	1	PQ533551
AV-3	Avian	*A. gazella*	-/+	-/+	G41	3	PQ581148
AV-4	Avian	*M. leonina*	-/-	-/-	-	-	-
AV-5	Avian	*A. gazella*	-/+	-/-	-	-	-
AV-6	Avian	*A. gazella*	+/-	+/-	O44	2	PQ581115
AV-7	Avian	*A. gazella*	-/-	-/-	-	-	-
AV-8	Avian	*A. gazella*	-/+	-/-	-	-	-
AV-9	Avian	*M. leonina*	-/+	-/-	-	-	-
AV-10	Avian	*M. leonina*	+/-	+/-	O48	2	PQ581116
AV-11	Avian	*A. gazella*	+/+	+/+	G49	1	PQ533552
					O49	2	PQ581117
AV-12	Avian	*A. gazella*	-/+	-/+	G50	1	PQ533553
AV-13	Avian	*L. weddellii*	+/-	+/-	-	-	-
AV-14	Avian	*L. weddellii*	+/-	+/-	-	-	-
AV-15	Avian	*M. leonina*	-/-	-/-	-	-	-
AV-16	Avian	*A. gazella*	-/-	-/-	-	-	-
AV-17	Avian	*A. gazella*	-/+	-/+	G55	1	PQ533554
AV-18	Avian	*A. gazella*	-/+	-/+	G56	2	PQ581113
AV-19	Avian	*A. gazella*	-/-	-/-	-	-	-
AV-20	Avian	*A. gazella*	+/-	+/-	O58	2	PQ581118
DE-1	Deception	*L. weddellii*	-/+	-/-	-	-	-
DE-2	Deception	*A. gazella*	-/+	-/+	G105	2	PQ581114
DE-3	Deception	*A. gazella*	+/-	+/-	O106	2	PQ581119
DE-4	Deception	*A. gazella*	+/-	+/-	O107	3	PQ581149
DE-5	Deception	*A. gazella*	-/-	-/-	-	-	-
DE-6	Deception	*A. gazella*	+/-	+/-	O109	2	PQ581120
DE-7	Deception	*A. gazella*	-/+	-/+	-	-	-
DE-8	Deception	*A. gazella*	+/-	+/-	O111	2	PQ581121
DE-9	Deception	*A. gazella*	-/+	-/-	-	-	-
DE-10	Deception	*L. weddellii*	+/-	+/-	O113	2	PQ581122
DE-11	Deception	*L. weddellii*	+/-	+/-	O114	4	PQ581158
DE-12	Deception	*A. gazella*	+/-	+/-	O115	3	PQ581150
DE-13	Deception	*L. weddellii*	+/-	+/-	O116	4	PQ581159
DE-14	Deception	*A. gazella*	+/+	+/+	G129	1	PQ533555
					O129	3	PQ581151
DE-15	Deception	*A. gazella*	+/-	+/-	O130	2	PQ581123
DE-16	Deception	*A. gazella*	+/-	+/-	O131	3	PQ581152
DE-17	Deception	*A. gazella*	+/-	+/-	O132	2	PQ581124
DE-18	Deception	*A. gazella*	+/-	+/-	O133	2	PQ581125
DE-19	Deception	*A. gazella*	+/+	+/+	O134	2	PQ581126
DE-20	Deception	*A. gazella*	+/-	+/-	O135	2	PQ581127
DE-21	Deception	*A. gazella*	+/-	+/-	O136	3	PQ581153
DE-22	Deception	*L. weddellii*	+/-	+/-	O137	2	PQ581128

DE-23	Deception	*L. weddellii*	+/-	+/-	-	-	-
DE-24	Deception	*A. gazella*	+/-	+/-	O139	2	PQ581129
DE-25	Deception	*A. gazella*	+/-	+/-	O140	2	PQ581130
DE-26	Deception	*A. gazella*	+/-	+/-	O141	3	PQ581154
DE-27	Deception	*A. gazella*	+/-	+/-	-	-	-
DE-28	Deception	*A. gazella*	+/-	+/-	-	-	-
DE-29	Deception	*L. weddellii*	+/-	+/-	O155	5	PQ581161
DE-30	Deception	*L. weddellii*	+/-	+/-	-	-	-
DE-31	Deception	*L. weddellii*	+/-	+/-	O157	2	PQ581131
DE-32	Deception	*L. weddellii*	+/-	+/-	-	-	-
DE-33	Deception	*L. weddellii*	+/-	+/-	O159	2	PQ581132
DE-34	Deception	*A. gazella*	+/-	+/-	O160	2	PQ581133
DE-35	Deception	*A. gazella*	+/-	+/-	O161	2	PQ581134
DE-36	Deception	*A. gazella*	+/-	+/-	O162	2	PQ581135
DE-37	Deception	*A. gazella*	+/-	+/-	O163	2	PQ581136
DE-38	Deception	*A. gazella*	+/-	+/-	O164	2	PQ581137
DE-39	Deception	*A. gazella*	+/+	+/-	-	-	-
DE-40	Deception	*A. gazella*	+/-	+/-	O166	2	PQ581138
DE-41	Deception	*A. gazella*	+/+	+/-	O167	3	PQ581155
DE-42	Deception	*A. gazella*	+/-	+/-	O168	2	PQ581139
DE-43	Deception	*L. weddellii*	+/-	+/-	O169	5	PQ581160
DE-44	Deception	*L. weddellii*	+/-	+/-	O170	2	PQ581140
DE-45	Deception	*L. weddellii*	+/+	+/-	-	-	-
DE-46	Deception	*M. leonina*	+/-	+/-	O172	5	PQ581162
DE-47	Deception	*A. gazella*	+/+	+/+	O173	2	PQ581141
DE-48	Deception	*A. gazella*	+/-	+/-	-	-	-
DE-49	Deception	*A. gazella*	+/-	+/-	-	-	-
DE-50	Deception	*A. gazella*	+/-	+/-	O176	2	PQ581142
DE-51	Deception	*A. gazella*	+/+	+/-	O177	2	PQ581143
DE-52	Deception	*A. gazella*	+/-	+/-	O178	2	PQ581144
DE-53	Deception	*A. gazella*	+/-	+/-	O179	2	PQ581145
DE-54	Deception	*A. gazella*	+/-	+/-	O180	2	PQ581146
DE-55	Deception	*A. gazella*	+/-	+/-	O181	3	PQ581156
DE-56	Deception	*A. gazella*	+/-	+/-	O182	3	PQ581157
DE-57	Deception	*A. gazella*	+/-	+/-	-	-	-
DE-58	Deception	*A. gazella*	+/-	+/-	O184	2	PQ581147
DE-59	Deception	*A. gazella*	+/-	+/-	-	-	-
DE-89	Deception	*A. gazella*	+/-	+/-	O89	6	PQ581164

*G: Genital sample; O: Oral sample
